# Supplementary material for: Echolocation-related reversal of information flow in a cortical vocalization network
Source: Nat Commun. 2022 Jun 25;13:3642. doi: 10.1038/s41467-022-31230-6 (PMC9233670; doi:10.1038/s41467-022-31230-6)
Supplement: Supplementary file 3 — Reporting Summary [file 41467_2022_31230_MOESM3_ESM.pdf]

nature research

Corresponding author(s): Francisco García-Rosales
Julio C. Hechavarria

Last updated by author(s): Apr 29, 2022

Reporting Summary

Nature Research wishes to improve the reproducibility of the work that we publish. This form provides structure for consistency and transparency in reporting. For further information on Nature Research policies, see our Editorial Policies and the Editorial Policy Checklist.

Statistics

For all statistical analyses, confirm that the following items are present in the figure legend, table legend, main text, or Methods section.

n/a	Confirmed	
☐	☒	The exact sample size (n) for each experimental group/condition, given as a discrete number and unit of measurement
☐	☒	A statement on whether measurements were taken from distinct samples or whether the same sample was measured repeatedly
☐	☒	The statistical test(s) used AND whether they are one- or two-sided<br>*Only common tests should be described solely by name; describe more complex techniques in the Methods section.*
☐	☒	A description of all covariates tested
☐	☒	A description of any assumptions or corrections, such as tests of normality and adjustment for multiple comparisons
☐	☒	A full description of the statistical parameters including central tendency (e.g. means) or other basic estimates (e.g. regression coefficient) AND variation (e.g. standard deviation) or associated estimates of uncertainty (e.g. confidence intervals)
☐	☒	For null hypothesis testing, the test statistic (e.g. F, t, r) with confidence intervals, effect sizes, degrees of freedom and P value noted<br>*Give P values as exact values whenever suitable.*
☒	☐	For Bayesian analysis, information on the choice of priors and Markov chain Monte Carlo settings
☒	☐	For hierarchical and complex designs, identification of the appropriate level for tests and full reporting of outcomes
☐	☒	Estimates of effect sizes (e.g. Cohen's d, Pearson's r), indicating how they were calculated

Our web collection on statistics for biologists contains articles on many of the points above.

Software and code

Policy information about availability of computer code

Data collection	Electrophysiological data collection was performed with a commercially available software: MC_Rack_Software (Multichannel Systems; version 4.6.2). Similarly, acoustic data collection was done using the Avisoft Recorder software (Avisoft Bioacustics, versions 4.2.8 and 4.3.01).
Data analysis	Data analyses were performed by means of custom-written scripts in MatLab (v. R2018b, and v. 8.6.0.267246 (R2015b)), Python (version 2.6 and 3.6), and R (RStudio v. 1.3.1073). Analyses relied partly on available toolboxes and packages, which are appropriately described in the Methods section of the manuscript. Packages used include Chronux (Matlab Toolbox, v. 2.12), and Brainstorm.

For manuscripts utilizing custom algorithms or software that are central to the research but not yet described in published literature, software must be made available to editors and reviewers. We strongly encourage code deposition in a community repository (e.g. GitHub). See the Nature Research guidelines for submitting code & software for further information.

Data

Policy information about availability of data

All manuscripts must include a data availability statement. This statement should provide the following information, where applicable:

- Accession codes, unique identifiers, or web links for publicly available datasets
- A list of figures that have associated raw data
- A description of any restrictions on data availability

A data availability statement is included in the manuscript: "The data generated during and/or analysed during the current study are available in the G-Node GIN repository, https://doi.gin.g-node.org/10.12751/g-node.q6xwhi/. Some data could not be uploaded due to its size; they are available from the authors upon request. Source data are provided with this paper."

Field-specific reporting

Please select the one below that is the best fit for your research. If you are not sure, read the appropriate sections before making your selection.

[x] Life sciences [ ] Behavioural & social sciences [ ] Ecological, evolutionary & environmental sciences

For a reference copy of the document with all sections, see nature.com/documents/nr-reporting-summary-flat.pdf

Life sciences study design

All studies must disclose on these points even when the disclosure is negative.

Sample size	We performed no statistical procedures to determine sample sizes. The sample size (number of paired penetrations) was chosen based on appropriate sample sizes from previous studies. The number of simultaneous penetrations (n = 30) in FAF and AC (penetration pairs were independent from one another) was chosen according to literature standards (e.g. Lakatos et al. (2013), Weineck et al. (2020)). The number of individual recordings in AC paired with electrical microstimulation of the FAF was determined in a similar way (n = 20). From a large group of detected vocalizations (n = 12494) produced by three bats, two subgroups of echolocation (n = 138) and unspecific communication calls (n = 734) were used. The calls in these groups were free from pre-vocal acoustic contamination. Each call was treated a single vocalization trial.
Data exclusions	Some of the calls were excluded from analyses. The reason for the exclusions was the need for a pre-vocal period without acoustic contamination. Acoustic contamination of this sort would have added confounding effects for the analyses. This was a pre-established criterion.
Replication	Spectral analyses described in the manuscript were performed with the Chronux Toolbox, which is based on a multi-taper approach (3 tapers). We also conducted other analyses (Matlab's built-in pmtm function and Welch's method) to verify that the consistency of the data derived from the spectra (one time). Part of the spectral data replicated and complemented a previous study from our laboratory (Weineck et al. (2020)). Transfer entropy analyses were conducted twice. On a first step, we conducted information transfer on non-paired pre-vocal and post-vocal periods (not published), which then were paired on a second re-analysis of the data (as described in the Methods). The patterns observed were qualitatively similar for both steps. In addition, we performed the second step several times (as randomization was involved) to verify that results were consistent across runs (3 times). All results obtained after randomization were verified several times, and data analyses were re-run once more during the review process. The main results of our study remained unaltered.
Randomization	Samples were not allocated in experimental groups, beyond the classification of vocalizations in echolocation and communication (also in low- and high-frequency communication). Randomization of the data was performed for the transfer entropy analyses, as described in the Methods. Further randomization was performed for analyzing data from electrical microstimulation experiments. Because subjects (i.e. individual animals) were not assigned into categories, randomization of test subjects does not apply.
Blinding	Classification of vocalizations in echolocation and communication was not performed blindly, as calls were manually curated in terms of their assignment into one or the other group. Experimenters were blind to what type of vocalizations animals produced within sessions. All assignments were done during offline pre-processing of the calls. Other forms of blinding are not relevant to our study.

Reporting for specific materials, systems and methods

We require information from authors about some types of materials, experimental systems and methods used in many studies. Here, indicate whether each material, system or method listed is relevant to your study. If you are not sure if a list item applies to your research, read the appropriate section before selecting a response.

Materials & experimental systems

n/a	Involved in the study	
x		Antibodies
x		Eukaryotic cell lines
x		Palaeontology and archaeology
	x	Animals and other organisms
x		Human research participants
x		Clinical data
x		Dual use research of concern

Methods

n/a	Involved in the study	
x		ChIP-seq
x		Flow cytometry
x		MRI-based neuroimaging

Animals and other organisms

Policy information about studies involving animals; ARRIVE guidelines recommended for reporting animal research

Laboratory animals	Animals used were adult bats of the species Carollia perspicillata (4 males; 1 female). As animals were captured from a large colony, the age of each individual could not be defined.
Wild animals	This study did not involve wild animals.

Field-collected samples	The current study did not involve samples collected in the field.
Ethics oversight	All experimental procedures were in compliance with European regulations for animal experimentation and were approved by the Regierungspräsidium Darmstadt (experimental permit #FU-1126 and FR-2007).

Note that full information on the approval of the study protocol must also be provided in the manuscript.
